# Effects of Acute Caffeine Ingestion on Morning Temperature, Mood, Reactive Agility and Cognitive Measures in Males: A Standardized Approach

**DOI:** 10.3390/nu18172771

**Published:** 2026-08-25

**Authors:** Ben J. Edwards, Magali Giacomoni, João P. S. Agulhari, Benoit Mauvieux, Samuel A. Pullinger, Sophie J. Vickery, Gillian M. Cook, James W. Roberts, Neil Chester

**Affiliations:** 1 Research Institute for Sport and Exercise Sciences, John Moores University, Liverpool L3 3AF, UK; j.agulhari@2022.ljmu.ac.uk (J.P.S.A.); samuel.pullinger@inspireinstituteofsport.com (S.A.P.); s.vickery@2022.ljmu.ac.uk (S.J.V.); g.m.cook@ljmu.ac.uk (G.M.C.); j.w.roberts@ljmu.ac.uk (J.W.R.); n.chester@ljmu.ac.uk (N.C.); 2 Laboratory Youth Physical Activity Sport and Health (J-AP2S), University of Toulon, 83130 La Garde, France; magali.giacomoni@univ-tln.fr; 3 Research Unity VERTEX 7480, UFR STAPS, University of Normandie, 14032 Caen, France; benoit.mauvieux@unicaen.fr; 4 Sport Science Department, Inspire Institute of Sport, Vidyanagar, Bellary 583275, India

**Keywords:** cognition, placebo effect, expectancy, diurnal variation, core temperature

## Abstract

**Background/Objectives**: We investigated whether ingestion of caffeine (~1 h before) was beneficial to subsequent morning (~07:30 h) mood, reactive agility, and cognitive measures in naïve-mild habitual caffeine consumers. **Methods**: A total of 150 recreationally active males were recruited, of whom 51 participated and completed six sessions as follows: (i) three familiarization sessions of all procedures at 12:00 h, and (ii) three experimental conditions. Participants completed caffeine and placebo trials under double-blind conditions, together with an open-label no-pill control trial using the stratified block randomization method based on baseline performance, which were counterbalanced in a crossover design into either caffeine (CAFF, 300 mg or 2.5–5.3 mg·kg^−1^ body weight), placebo (PLAC), or no-pill control (NoPill), both ingested at ~06:30 h. For each experimental session, on arrival at the laboratory, questions on sleep, mood states, and caffeine withdrawal were asked. A battery of cognitive performance tests was then administered (trail-making test, Rey’s auditory verbal learning test, and Stroop word–colour interference test). At 30 min of rest and after a 5 min warm-up on a treadmill with associated stretches, rectal and mean skin temperatures (T_r_ and T_sk_) were measured. Thereafter, two 90 s reactive agility tests (RATs) were undertaken, with average movement time(s) and contact number per light recorded. Data were analyzed using a general linear model with repeated measures, but the RAT measures were run as ANCOVA with mass used as a covariate. **Results**: The battery of cognitive tests showed no significant main effects for condition, whether the body mass of the participants was considered or not. There were no significant main effects of the experimental condition for RAT variables. There was a main effect for “light position”, for mean movement time values (*p* < 0.001, η^2^_p_ = 0.508), and cognitive–motor efficiency (contacts·s^−1^, *p* < 0.001, η^2^_p_ = 0.582), where pairwise analysis showed mean movement times as higher, and cognitive–motor efficiency values indicating a lower rate of successful responses during movement for lights 1 and 5 (the furthest from the start point corresponding to light 3), rather than lights 2, 3 and 4. There were no significant interactions for condition and light for any of the variables. **Conclusions**: Early morning ingestion of 300 mg of caffeine had no effect on the battery of cognitive or reactive agility test measures in the population of naïve-mild habitual caffeine consumers. Caffeine ingestion had no effect on T_r_, T_sk_, mood, or caffeine withdrawal symptom scores, as well as tiredness or alertness, with or without consideration for body mass.

## 1. Introduction

Athletes are frequently required to perform during morning hours due to tournament scheduling, practicalities associated with training demands, and environmental considerations [1,2]. Early morning is associated with circadian-related reductions in physiological function, as well as decreases in gross muscular and overall physical performance capacity. Cognitive performance is also an important determinant of athletic success, although the magnitude of diurnal variation appears to depend on the specific cognitive domain assessed. Reported differences range from 9.0 to 34.2% for reaction time, 7.3% for alertness, and 7.8 to 40.3% for attention [3]. Although the relative contributions of circadian (approximately 24 h), ultradian (<24 h), and/or time-since-awake factor (homeostatic drive) processes remain unclear, the type of cognitive function being assessed appears to be an important determinant of whether performance is optimal in the morning, evening, or afternoon [3]. Furthermore, subjective negative mood states, particularly fatigue, and levels of motivation, which are essential elements for performance on tasks requiring higher cognitive function, are less favourable in the morning than in the evening [2].

Approaches used to characterize and classify cognitive performance in clinical neuropsychology typically refer to domains of cognitive performance. Methods to conceptualize cognitive ability domains include the following: (i) Classification by the general process involved. This includes sensation, perception, motor skills and construction, attention and concentration, executive functioning, processing speed, and language/verbal skills [4]; (ii) regional brain functions originating from the frontal lobe, temporal lobe, parietal lobe, hippocampus, or other structures; and (iii) a hierarchical organizational structure, based on the complexity of the operations. They are often referred to as top-down versus bottom-up, where basic sensory and perceptual operations are least complex (where minimal higher-level processing is required), and reasoning and problem solving involve the co-ordination of multiple sensory, perceptual, and attentional factors and are most complex (referred to as executive functioning). Within each domain there are typically subdomains, which refer to component ability processes within the larger constructs. Inconsistencies in interpretation exist, mostly in broad domains that may include multiple component processes. Whether these processes belong in more general domains (executive functioning) or a simpler domain (processing speed) is often unclear.

Caffeine (1,3,7-trimethylxanthine) enhances arousal and reduces fatigue by antagonizing adenosine receptors within key brain regions such as the hypothalamus, basal forebrain, and reticular activating system, thereby suppressing sleep-promoting pathways and increasing neurotransmitter activity (e.g., dopamine and noradrenaline) involved in attention and wakefulness [5,6,7]. This results in improvements in strength measures, as well as time-trial and repeated sprint ability performance [8,9,10,11,12,13]. The literature suggests that caffeine’s strongest morning effects occur in alertness, vigilance, sustained attention, reaction time, and subjective energy [3]. Standard psychometric tests of cognition, as well as reactive agility, e.g., reaction times, decision-making accuracy, executive control, executive function (inhibitory control, cognitive flexibility/set shifting), verbal learning and memory, and processing speed, collectively cover four major domains: motor skills, executive function, processing speed, and memory. Apart from the Psychomotor Vigilance Task (PVT), which measures attention and vigilance, these are among the cognitive functions most frequently examined in circadian and caffeine research. The observed effects of caffeine on mood, agility, and cognitive performance may be confounded by methodological limitations. These include inconsistent dosing and timing protocols, a lack of placebo-controlled (NoPill) conditions, and inadequate familiarization procedures [14,15,16]. Moreover, the absence of power-based sample size calculations and poor standardization of habitual caffeine intake may further compromise study validity [17]. Inadequate control of chronobiological variables, such as chronotype, time-of-day, season, and sleep quality, may also obscure the detection of caffeine’s effects on morning reactive agility and cognitive performance [16]. Previous methodological assessments have indicated that the most common sources of bias in individual studies arise from bias in outcome measurements and bias in the selection of reported results, highlighting a lack of well-controlled investigations in this area [18]. Consequently, recent systematic reviews have emphasized the need for more rigorous methodological approaches when examining daily variations in exercise and cognitive performance, and when evaluating the effects of interventions such as caffeine [19,20].

Therefore, the aims of this study were to use a well-controlled and scientifically rigorous protocol to assess the effects of 300 mg of caffeine (CAFF) vs. a placebo (PLAC) vs. a control no-pill (NoPill) condition on reactive agility and cognitive performance (trail-making test, Rey’s auditory verbal learning test, and Stroop word–colour interference test). We chose a population of naïve-mild habitual caffeine consumers < 150 mg to reduce the effects of caffeine withdrawal symptoms in the non-caffeine condition on performance, as well as maximizing the caffeine effects at the 300 mg dose administered. A secondary aim was to measure core body and mean skin temperatures (T_r_ and T_sk_), as well as mood withdrawal symptoms, appetite, and satiety. We hypothesized that caffeine would improve morning agility and cognitive performance compared to the other conditions (NoPill and PLAC) whilst reducing negative mood states and tiredness.

## 2. Materials and Methods

### 2.1. Participants

This was a secondary analysis from a broader programme of research investigating caffeine effects on morning performance, from which mood, sleep, and caffeine withdrawal symptoms data have been previously published [11,12,13]. Data from the cognitive test battery have been published for a subset of participants (n = 17) [11], whereas the reactive agility data have not been reported previously. We followed the standardized reporting guidance outlined in the CONSORT extension for crossover trials [21] (checklist is attached in Supplementary Materials; Figure S1).

Fifty-one adult males, as identified from biological sex and gender, who were classified as “recreationally active” by the “Participant classification framework” [22], participated in the present study. Physical characteristics, baseline caffeine intake, and daily food intake of the participants are presented in Table 1. Participants completed a 7-day habitual food/fluid diary and weighed food intake (Table 1). The food diaries were analyzed with Nutritics^®^ analysis software (Version 6.23; Nutritics Ltd., Dublin, Ireland); this process was conducted by an SENR-registered Sports and Exercise Nutritionist. Key information such as daily calories, macro- and micronutrient intake, as well as levels of caffeine and water intake, were compared against recommended daily intake amounts [23]. This population consumed more protein, carbohydrates, Vitamin B_6_, and water, but fewer daily calories and magnesium than recommended (Table 1). These levels of carbohydrates and protein reflect the intake needed for the demands of exercise and recovery. We originally opened participation to females, but no females volunteered. All participants were recruited from the University of Sport Science Department student population. Components of this study were registered retrospectively on ClinicalTrials.gov (registration numbers: NCT07466628, NCT07469852 and NCT07466732), approved in November 2024 and March 2026, respectively. At the time of study initiation, the research team did not realize that prospective registration was required for a non-clinical trial of investigational medicinal products (CTIMP) nutrition-based crossover study involving healthy participants and sporting performance outcomes. Upon becoming aware of journal and transparency requirements, this study was registered to ensure public accessibility and accountability. The study protocol, eligibility criteria, intervention conditions, and primary and secondary outcome measures were defined prior to participant recruitment and were not modified following study commencement. No changes were made to the study design or outcomes after registration. Due to equipment availability, only 27/51 participants were tested for RAT assessment.

Inclusion criteria were healthy males (18–30 years) who were injury-free; these were participants who habitually retired between 22:00 and 23:30 h and rose at 06:00–07:30 h, and who agreed to retire to bed at 22:30 and rise at 06:30 h for the duration of the study. None of the participants were receiving any pharmacological treatment (including non-steroidal anti-inflammatory drugs, NSAIDs) throughout the study period. Habitual caffeine consumption was assessed using the caffeine consumption questionnaire (CCQ) [24], and those having a consumption of ≥150 mg·day^−1^ were excluded, leaving only naïve-mild users (1.27± 0.67 mg·day^−1^) [25]. A total of 150 students were screened, of whom 55 met the criterion of habitual caffeine consumption <150 mg·day^−1^. Four participants subsequently withdrew, leaving a final sample of 51 volunteers. These individuals were allocated to dates and schedules of testing at their convenience; in practice, participation was completed over a 3-week period (see Supplementary Materials for CONSORT flow diagram; Figure S2 [21]). Furthermore, based on a self-reported training diary completed over a 2-week period, participants reported no preference for training at a particular time of day. Exclusion criteria included depressed mood (from the Beck depression inventory [26]); poor sleep quality (a Pittsburgh sleep quality index global score > 5 [27]); recent shift work, travel across multiple time zones, ‘extreme’ chronotype (assessed via the Composite Morningness Questionnaire [28]), and risk factors or symptoms of cardiovascular disease. Through interviews, it was established that the volunteers had minimal knowledge of the effects of time-of-day or time-since-sleep on human performance. Verbal explanation of the experimental procedure was provided to everyone; this included the aims of the study, the possible risks associated with participation, and the experimental procedures that were to be utilized. Any questions were answered. Individuals then provided written, informed consent before participating in the study. The experimental procedures were approved by the Human Ethics Committee at Liverpool John Moores University (ETHICS CODES: M24_SPS_4442; 251127LJMUSPSREC93 and 08/SPS/030). This study was conducted in accordance with the ethical standards of the journal and complied with the principles of the Declaration of Helsinki.

### 2.2. Experimental Design

**Figure 1:**
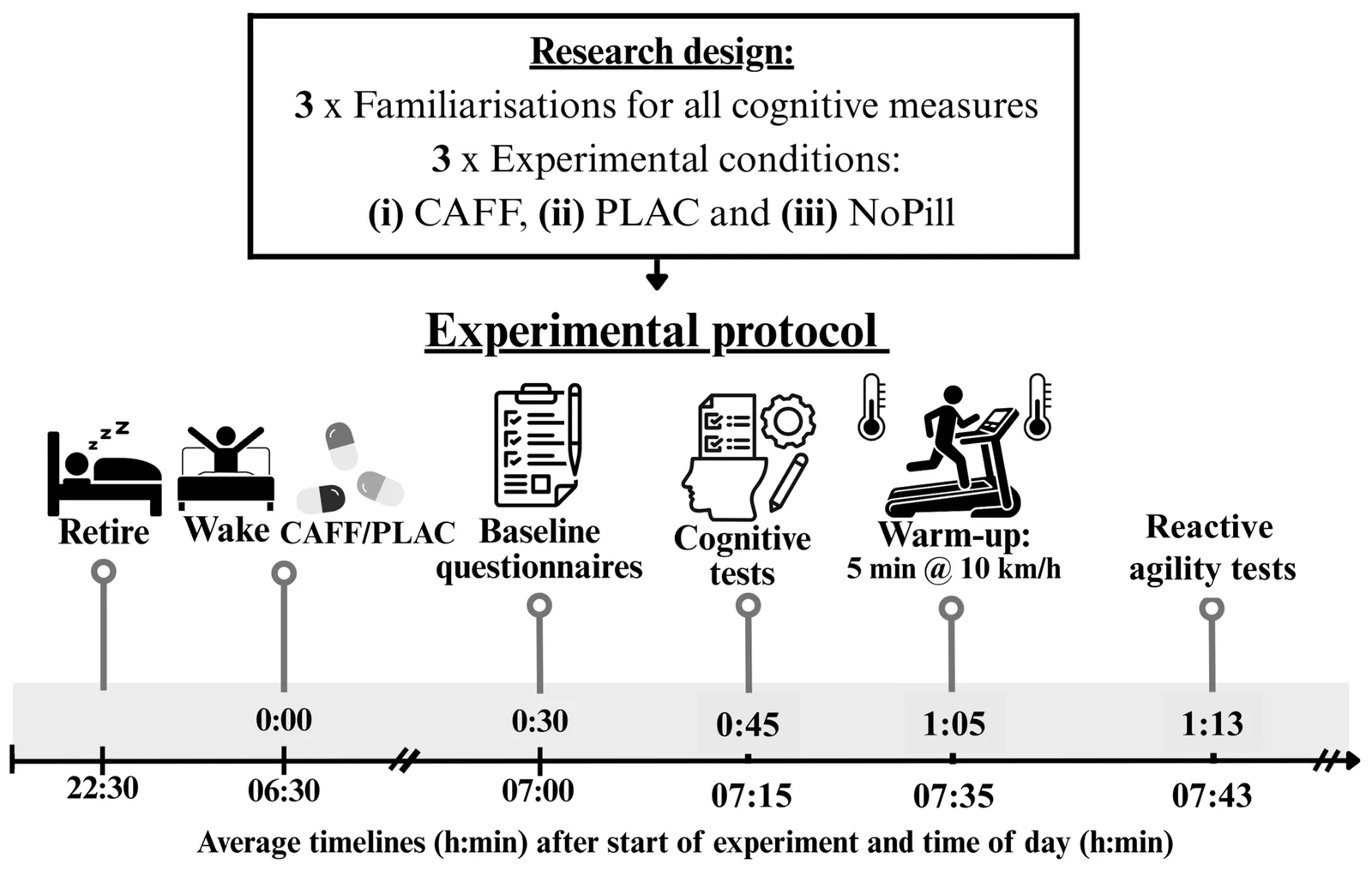
Schematic representation of the research design and experimental protocol undertaken by participants in the investigation. The first/top timeline starts from waking, when pills were taken; 30 min later, participants arrived at the laboratory, through to the completion of the reactive agility tests. The bottom timeline starts from retiring and ends with finishing the testing. In both timelines, the points are 15 min earlier or later, depending on individual scheduling. The thermometer represents rectal and mean skin (T_r_ and T_sk_) temperature measurements. The clipboard indicates where subjective resting ratings through questionnaires (Q’s) and cognitive tests were undertaken. Participants were asked to identify their condition just before the warm-up and immediately after the reactive agility test.

The participants visited the physiological laboratory at JMU (Liverpool, UK) on six occasions, with environmental conditions comprising a wet-bulb globe temperature of 13–17 °C, dry temperatures of 19–21 °C, 26–44% humidity, a barometric pressure of 990–1022 mmHg, and an ambient light of 750 lux. Each participant completed three familiarization sessions. The remaining sessions consisted of three experimental sessions, separated by at least 72 h to allow sufficient recovery (Figure 1). All the trials were performed with only one participant at a time, but with a staggered start so one participant came in at 06:45, the next at 07:00 h, and the last for that morning’s session at 07:15 h. This scheduling for participants was kept for all sessions and resulted in bedtimes and wake times, as well as pill ingestion, being the same or 15 min earlier or later than the example given in Figure 1 or in the text below. The experimental part comprised three experimental trials: (a) NoPill control (NoPill), (b) caffeine (300 mg, CAFF), and (c) placebo (maltodextrin, PLAC). Depending on scheduling, participants retired the previous night at around 22:30 h, woke up at ~06:30 h, and arrived at the laboratory at ~07:00 h in a fasted state. For the CAFF and PLAC conditions, participants also ingested 3 × size 00 capsules upon waking (~06:30 h), prior to arrival at the laboratory. Participants were instructed to avoid training or intense physical activity 48 h prior to any of the visits; otherwise, participants maintained their normal daily routines. Further, for the 24 h period before the experimental conditions, participants were asked to replicate their diet, feeding pattern, and macro/micronutrient contents, but in the absence of alcohol and caffeine intake from their habitual 7-day dietary recall. This balanced diet was based on foods that they normally consumed in their diet, but that were matched to RDA values and prescribed by a member of the research team [23]. Adherence to the diet and abstinence from caffeine, alcohol, and exercise, as well as a sleep schedule, were verbally asked on arrival, and responses were recorded by an experimenter. Compliance was high, with all but two participants adhering to the protocol (due to illness); these individuals were not tested and repeated the condition later in the month when they were not ill. During this time, researchers asked about any negative side effects from the condition; no participant reported any adverse effects. In total, the three caffeine capsules contained 300 mg of caffeine anhydrous, and the remaining space was occupied by maltodextrin (Applied Nutrition LTD., Knowsley, UK); placebo capsules were made in the department and contained maltodextrin (~1.55 g, Sport Supplements Ltd. t/a Bulk^TM^, Colchester, UK). Researchers and participants were blinded to the supplement schedule, and pills were provided in a plastic bottle with instructions to consume with water. Both caffeine and placebo capsules were lightly dusted with maltodextrin to create a similar taste; both had similar weight (0.8 g/capsule) and were of 00 size. At the end of the experiment, the order of treatment was revealed to the researchers by an author (BE).

Participants were allocated to groups using a two-stage procedure. First, they were sorted into three matched performance blocks (high, medium, and low) based on the number of successful light contacts achieved during the reactive agility test in the third familiarization session. Within each performance block, participants were then randomly assigned to one of the six treatment sequences required for counterbalancing the three experimental conditions (ABC, ACB, BAC, BCA, CAB, and CBA). Randomisation was performed by the lead author (B.J.E.), who selected sequence numbers from a box. This procedure ensured both performance matching across groups and treatment-order counterbalancing. Following allocation to an initial condition, the potential influence of learning effects was minimized using a counterbalanced crossover design [16].

This study was conducted between November and February (autumn to winter in the UK), during which morning sunlight exposure prior to laboratory arrival was limited to <80 lux. During the study period, sunrise occurred between 07:41 and 08:03 h (accounting for atmospheric refraction). Recruitment commenced immediately following ethical approval, and no participant reported any adverse events or harm during the study.

### 2.3. Protocol and Measurements: Familiarization Session

Participants completed three familiarization sessions involving the reactive-agility, cognitive assessments, and questionnaires before being considered ready to participate in the study. A coefficient of variation value of ≤10% between the third and second assessments was used as a criterion for adequate familiarization, in alignment with the recommendations given by Atkinson and Nevill [29]. For RAT total contacts, TMT-A; TMT-B; RAVLT—distractions; RAVLT—retention score; Stroop colours, not-words; and words, not colour scores, coefficients of variance (%), percent changes, and *p*-values demonstrated acceptable stability between sessions (CV = 9.6, 8.2, 7.2, 8.6, 6.3%; % change = 7.0, 5.9, 3.9, 7.1, 7.2%; *p* > 0.05). Familiarization sessions were conducted at 12:00 h over a three-week period and were completed one week before the study commenced to minimize learning effects. This testing time was selected because it was convenient for the participants and avoided the need for three additional early-morning laboratory visits that may have disrupted their sleep patterns. To minimize potential learning and memory effects, participants were provided with different versions of the Stroop test, trail-making test (Parts A and B), and Rey auditory verbal learning test during familiarization sessions. Participants arrived 30 min before testing and rested in a seated position to minimize the influence of prior physical activity. Familiarization sessions were completed in the following order: (i) the cognitive test battery and (ii) the reactive agility test.

(i) Cognitive battery of tests

The trail-making test (TMT; parts A and B): The TMT is a widely used assessment of processing speed, sequencing ability, visual search, and visuomotor skills. Both parts of the TMT consist of 25 circles distributed over a sheet of A4 paper. In Part A, the circles are numbered 1–25, and the participant is instructed to draw lines to connect the numbers in ascending order. In Part B, the circles include both numbers (1–13) and letters (A–L), and the participant is instructed to draw lines to connect the circles in an ascending pattern but with the added task of alternating between numbers and letters (i.e., 1–A–2-B–3–C, etc.). In both parts, participants were instructed to connect the circles as fast as possible, without lifting the pencil from the paper. If an error is made, this is pointed out immediately, and the participant is allowed to correct it. During the test, completion time was measured for each part, with longer completion times indicating poorer performance [30]. In addition to completion times for Parts A and B, the difference in time between Part A and Part B completion times (B-A) was also calculated as an index of task-switching ability and cognitive flexibility.

The Rey auditory verbal learning test (RAVLT): The RAVLT is a neuropsychological assessment designed to evaluate verbal learning and memory in individuals aged 16 years and older [31]. It can be used to assess the nature and severity of memory dysfunction and to monitor changes in memory performance over time [31]. The test is employed as a list-learning paradigm in which the volunteer hears a list of 15 nouns (list A) and is asked to recall as many words as possible. The number of correctly recalled words and the number of words that were given by the participant but that are not on the list (intrusions) are noted. This procedure is repeated for a total of five learning trials. Following the fifth trial, a second list of 15 nouns (List B; interference list) is presented and recalled in the same manner. Immediately after recall of List B, participants are asked to recall the original List A, with the number of correctly recalled words and intrusions again recorded. Outcomes derived from the RAVLT and included in the analysis were total recall score, number of intrusions (distractions), and retention [31].

The Stroop word–colour interference test [32,33]: The Stroop test assesses inhibitory control underlying selective attention and decision-making abilities. Participants were filmed verbally responding to words or colours for 45 s as quickly as possible over four separate sheets. The first sheet had names of colours (henceforth referred to as colour-words, e.g., red, *blue*, *yellow*, *black*, *green*) printed in black ink that participants read out (naming word test, W). The second sheet had colour-words printed in corresponding colour ink (e.g., a *yellow* colour-word printed in yellow ink), and participants responded to the colour ink (naming colour test, C). The third sheet had colour-words printed in non-corresponding colour ink (e.g., a *yellow* colour-word printed in red ink), and participants responded to the colour ink (naming the colour of the word test, CW). The fourth sheet had the same colour-words printed in non-corresponding colour ink presented in reverse order to the third sheet, and participants read out the colour-word (naming of the word that is not in the colour test, WC). Importantly, the W (first sheet) and C (second sheet) test sheets acted as a baseline to the WC (fourth sheet) and CW (third sheet) test sheets, respectively. The raw data were analyzed for the number of errors (indicating pacing/speed accuracy in 45 s) and interference scores (indicating efficiency of the inhibitory function). For the naming of colours, the interference score was based on the correct answers in C and CW [34] and calculated as follows: [(C − CW)/(C + CW)] × 100. In a similar vein, for the naming of colour-words, the interference score was based on the correct answers in W and WC and calculated as follows: [(W − WC)/(W + WC)] × 100.

(ii) Reactive Agility Test (RAT)

**Figure 2:**
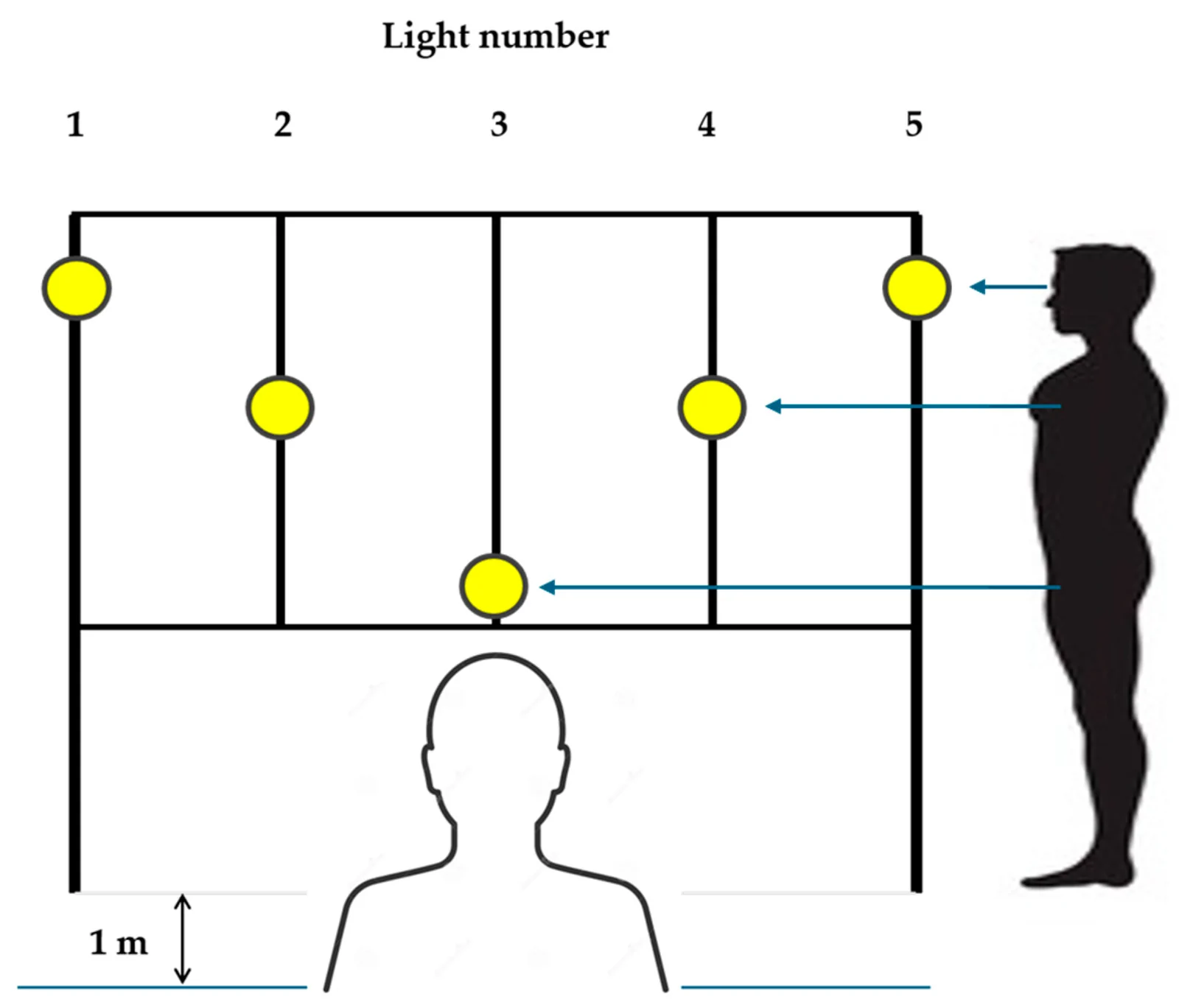
Diagram of the RAT, with participants located 1 m from the frame directly opposite light 3. Lights 1 and 5 were positioned roughly at eye level, 2 and 4 at shoulder level, and 3 at waist level.

The agility test was performed using a fixed frame consisting of five Witty SEM lights (Microgate, Bolzano, Italy) arranged in a V-shaped formation (See Figure 2). From left to right, light 1 was positioned at head height, light 2 at shoulder height, light 3 centrally at torso height, light 4 at shoulder height, and light 5 at head height. Participants stood behind a marked line positioned approximately 1 m parallel to the apparatus opposite light 3. Lights 1 and 5 were diagonally ~1.57 m from the starting point, and 2 and 4 were 1.47 m. During the test, a single light illuminated green at random; participants were instructed to react as quickly as possible by placing their hand directly in front of the activated light. Physical contact with the light was not required. Following each response, participants returned behind the marked line before reacting to the next stimulus. The two lights positioned on the left side of the apparatus were responded to by using the left hand; the two lights on the right side were responded to by using the right hand. Participants were required to respond to the central light with their dominant hand. Responses performed using the incorrect hand were recorded as errors. The test duration was 90 s and was completed twice, with the highest contacts (performance volume) retained for analysis. Reaction time could not be measured; therefore, where movement time is mentioned in the text, this is the combined reaction time plus the time taken to reach the target light, which was at different distances from the starting point (1.00, 1.47, and 1.57 m). Performance outcomes included the movement time for each light, cumulative movement time, movement time variability (SD), number of lights completed within 90 s, and total errors as a function of light, which were labelled sequentially from left to right as lights 1–5. (1) Total contacts (overall performance) or the total number of lights successfully reached/touched in 90 s; where higher represents a better overall performance. This task reflects the combined influence of reaction, decision-making, movement speed, and task execution capacity and is commonly used as a primary outcome in light-based reactive agility systems [35]. (2) Mean movement time (speed) is the sum of movement times divided by the total contact number, where lower values indicate faster movement performance. In this measure, movement time is a fundamental component of reactive agility and provides an index of physical execution speed independent of the total work completed [36]. (3) Cognitive motor efficiency was calculated as successful contacts divided by the total movement time (contacts·s^−1^), with higher values indicating a greater rate of successful responses during movement. This metric incorporates both motor speed and task success and has been proposed as a practical measure of perceptual motor efficiency [37]. (4) Variability of Movement Time [Coefficient of variation (% CV)]; this is calculated by using the SD of total movement time divided by the mean movement time, multiplied by 100 for each light. The coefficient of variation normalizes variability relative to mean performance and is frequently reported in agility and reactive-performance research when evaluating consistency and reliability [29,37]. A participant may achieve a fast mean movement time while exhibiting substantial inconsistency across trials; therefore, variability provides complementary information to average speed. Lower values indicate more consistent performance. Hand dominance for physical tasks such as throwing a ball and racket sports was recorded for later analysis. Unfortunately, due to equipment availability between the fourth week in January and the end of February, only 27 of the 51 participants completed the reactive agility test (RAT). The stratified block randomization method based on baseline performance, which was counterbalanced in a crossover design, ensured that the subpopulation from the initial sample had high, medium, and low performers randomly allocated into the crossover design. Baseline characteristics, including age, height, body mass, habitual caffeine intake, sleep quality, athletic level, and habitual sleep duration, were similar between included and excluded participants, suggesting a low risk of selection bias (age 21.9 ± 3.1 vs. 21.0 ± 1.2 years, height 178.0 ± 7.2 vs. 180.0 ± 8.3 cm, mass 83.9 ± 17.2 vs. 82.9 ± 15.3 kg, caffeine intake 101 ± 45 vs. 102 ± 51 mg·day^−1^, Pittsburgh sleep questionnaire 3.1 ± 1.1 vs. 3.3 ± 0.9, athletic levels 2.1 ± 1.0 vs. 2.1 ± 0.7, and hours slept habitually 8.2 ± 0.5 vs. 8.5 ± 0.7 decimal h).

### 2.4. Experimental Protocol and Measurements

The experimental sessions took place a week after the last familiarization, with 72 h recovery between the three experimental conditions (i.e., CAFF, PLAC, and NoPill). Participants arrived at the laboratory 5 min before the start of the test and were asked to insert a soft, flexible rectal probe (mini-thermistor, Grant Instruments (Cambridge) Ltd., Shepreth, Cambridgeshire, UK) approximately 10 cm beyond the external anal sphincter. Participants then rested in a supine position for 30 min to assess resting rectal temperature (T_r_). Skin temperature (T_sk_) was assessed simultaneously by skin thermistors (Grant Instruments (Cambridge) Ltd., Squirrel 2010 series, Cambridgeshire, UK), which were placed at four locations on the left side of the body (chest [ch], forearm [f], thigh [th] and calf [ca]). The average of the last 5 min of the 30 min resting period was recorded for resting T_r_ and T_sk_ temperatures. Weighted mean T_sk_ was calculated using the equation T_sk_ = (0.34 × T_ch_) + (0.33 × Tth) + (0.18 × T_ca_) + (0.15 × T_f_), by Ramanathan [38]. Participants, whilst resting, completed the profile of mood states questionnaire (POMS [39]) and their subjective rating of sleep (using sleep questions from the Liverpool Jet Lag Questionnaire [40]. Lastly, caffeine withdrawal symptoms were assessed using a caffeine withdrawal questionnaire [41] and hunger and satiety [42]. Fifteen minutes into the resting period, participants completed the battery of cognitive tests, which took ~20 min. Volunteers then warmed up on a treadmill for 5 min at 10 km.h^−1^, completed a series of stretches (for ~3 min), and then undertook the 90 s reactive agility test. Participants were asked in conditions CAFF and PLAC, just before the warm-up and after the reactive agility test, to state which condition they thought they were in.

### 2.5. Statistical Analysis

The main research variables in the present study were the battery of cognitive tests and the agility test. We took d = 0.40 as a moderate/conservative planning effect size, where caffeine ingestion has previously been shown to have Cohens d effect sizes (ES) on reactive agility (sprint time, total time, and decision time), Stroop measures (interference time and errors) and RAVLT total recalls of 1.92, 3.99, and 5.13 (η^2^_p_ = 0.480, 0.799, 0.868) [43], 0.49, 0.59 [44], and 1.06 (η^2^_p_ = 0.22), respectively [11]. Forty-one participants were estimated to provide 80% statistical power (*p* < 0.05), detecting a significant difference between conditions (NoPill, CAFF, and PLAC) with an effect size of 0.4 for a repeated analysis of variance (ANOVA). Therefore, we recruited 55 participants to allow for dropouts, with 4 not completing the experiment due to injury. The sample size estimation was performed using software G*Power 3.1 [45]. Data were analyzed using the Statistical Package for Social Sciences version 31 (SPSS, Chicago, IL, USA). All data were checked for normality by creating three pairwise differences: caffeine—NoPill, placebo—NoPill, and caffeine—placebo. Normality plots were conducted with tests considering Shapiro–Wilk results, as well as visually interpreting Q–Q plots and the presence of extreme outliers. In all cases, the normality of the difference scores was assessed using Q–Q plots and the Shapiro–Wilk test. In all but one case, visual inspection indicated that the difference scores were approximately normally distributed, with no substantial outliers. However, for the cognitive agility test, the Shapiro–Wilk test was significant, but the Q–Q plots looked reasonably straight, and there were no major outliers; therefore, the repeated-measures ANCOVA was still employed. To double-check the appropriateness of this analysis, data were log-transformed to make all values positive, and the ANCOVA was re-run. General linear models were used to analyze all measurements collected. Repeated-measures ANCOVA was performed with Condition (3 levels) as the within-subject factor and body mass as a covariate, where only one measure was taken for each condition. For variables measured more than once during the condition (such as pre and post), a repeated-measures ANCOVA was performed with (i) Condition (3 levels), (ii) Measure (2 levels), and (iii) interactions of Condition by Measure as the within-subject factors and body mass as a covariate. For the reactive agility test, repeated-measures ANOVA was performed with Condition (3 levels), Measure (5 levels), and (iii) interactions of Condition by Measure as the within-subject factors. The general linear model approach assumes a compound symmetric covariance structure, equivalent to the sphericity assumption. Sphericity was assessed using Mauchly’s test and, where violated, Greenhouse–Geisser (ε < 0.75) or Huynh–Feldt (ε > 0.75), and main effects were tested using Bonferroni pairwise comparisons with corrected degrees of freedom, and *p*-values were reported. There were no missing data; therefore, no cases were excluded from the repeated-measures analysis, as the GLM repeated-measures procedure requires complete data across all levels of the within-subject factor. Linear mixed-effects models were considered; however, because all participants provided complete data and the study design was balanced, a repeated-measures ANCOVA was deemed appropriate. All data are presented as means ± standard deviations (SD), unless stated otherwise. Significance was set at *p* ≤ 0.05. Partial eta squared values (η^2^_p_) were reported as the effect sizes, with values of 0.01, 0.06, and 0.14 corresponding to a small, medium, or large effect, respectively [46]. Ninety-five percent confidence intervals (95% CI) and mean differences between pairwise comparisons are reported where suitable.

## 3. Results

### 3.1. Rest and Post Warm-Up Variables

There were no significant main effects of the experimental condition for T_r_ and T_sk_ values, whether corrected for mass or not (*p* > 0.05, η^2^_p_ = 0.011 or 0.015; and η^2^_p_ = 0.078 or 0.098; Table 2). There was a significant main effect for “rest to post warm-up”, where T_SK_ values decreased (Δ0.88 °C, 95% CI = 0.78 to 0.99 °C, *p* < 0.001; η^2^_p_ = 0.834). This effect for T_SK_ was not significant when mass was considered (*p* = 0.836). T_r_ values did not show a change from rest to after the warm-up with and without considering mass (*p* > 0.05). There were no significant interactions; T_r_ and T_sk_ profiles for Condition from pre to post did not differ, regardless of considering body mass (*p* > 0.05, Table 2).

### 3.2. Hunger and Satiety Scales

There was no significant main effect of experimental condition on any of the hunger scales such as hunger, fullness of stomach, desire for savoury food, desire for sweet food, physical tiredness, sleepiness/drowsiness, energy/alertness, or lethargy/sluggishness (*p* > 0.05; Table 2). This was regardless of consideration for body mass or not.

### 3.3. Effectiveness of Counterbalancing and Response to Reactive Agility Test (RAT)

Data for movement time and number of contacts for the three conditions were reordered to first, second, and third sessions to investigate if the counterbalancing was effective. There was no significant effect of order for movement time and number of contacts (*p* = 0.525 and *p* = 0.676; η^2^_p_ = 0.024 and η^2^_p_ = 0.014, respectively).

**Figure 3:**
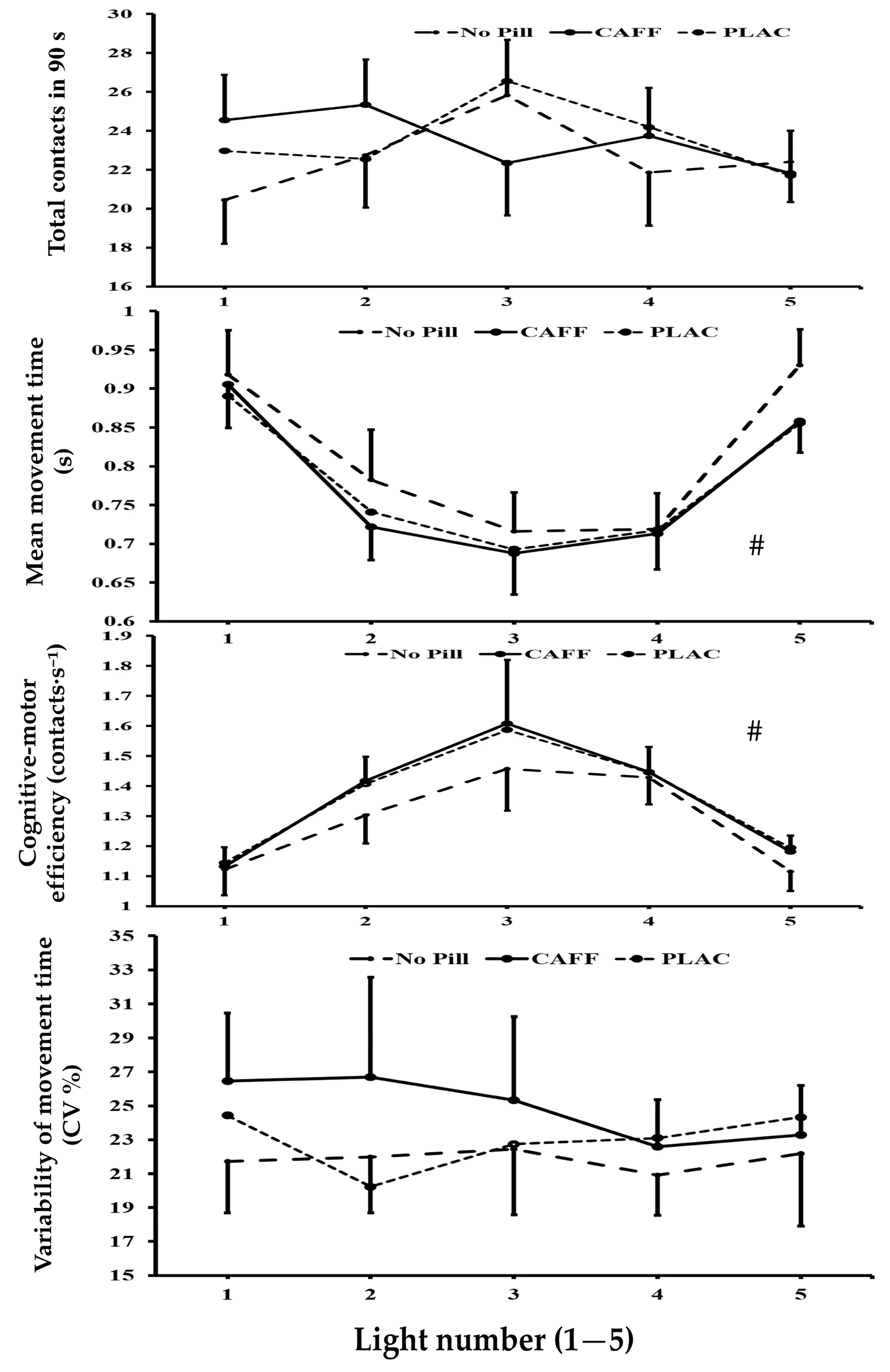
Mean (95% CI) values for the reactive agility test variables [Total contacts in 90 s, Mean movement time (s), Cognitive-motor efficiency (contacts·s^−1^), and Variability of movement time (CV %)] for the three conditions (NoPill, CAFF, and PLAC) by light number (n = 27). #—denotes main effect for light number.

There were no significant main effects of experimental condition for RAT variables (Table 2). There was a main effect of “light position” for mean movement time values (*p* < 0.001, η^2^_p_ = 0.508; Table 2), and cognitive-motor efficiency (contacts·s^−1^, *p* < 0.001, η^2^_p_ = 0.582; Table 2). Pairwise analysis showed that the mean movement times were higher and cognitive-motor efficiency values indicated a lower rate of successful responses during movement for lights 1 and 5 (the furthest from the start point corresponding to light 3) than for lights 2, 3, and 4 (Table 2, Figure 3). There were no significant interactions between condition and light for any of the variables.

### 3.4. Effectiveness of Counterbalancing and Response to Cognitive Function Tests

Data for TM-A; TM-B; word, not colours—number tests; colours, not words—number; word interference; colour interference; RAVLT—total number; RAVLT—distraction; and RAVLT—retention tests for the three conditions were reordered to first, second, and third sessions to investigate if counterbalancing was effective. There was no significant effect of order for any of these variables (*p* > 0.05; η^2^_p_ ≤ 0.035).

There were no main effects of condition for any of the battery of cognitive tests with or without consideration for body mass (Table 3).

### 3.5. Resting Profile of Mood States, Caffeine Withdrawal, and Sleep Questionnaires

There was no significant effect of condition in any mood states and sleep questionnaires, where there were no differences between NoPill, CAFF, and PLAC conditions (Table 4).

## 4. Discussion

The aims of this study were to assess the effects of 300 mg of caffeine (CAFF) vs. a placebo (PLAC) vs. a control no-pill (NoPill) condition on reactive agility and cognitive performance, as measured by the trail-making test, Rey’s auditory verbal learning test, and Stroop word–colour interference test. We selected a population of naïve-mild daily caffeine consumers (<150 mg·day^−1^) to minimize the effects of caffeine withdrawal in the non-caffeine conditions and to maximize the potential effects of the 300 mg caffeine dose administered. In the current protocol, there were no order effects for either the battery of cognitive tests or the agility test; therefore, potential residual learning effects were considered low (*p* > 0.05; η^2^_p_ ≤ 0.035). Participants showed a poor ability to correctly guess whether they had received caffeine or a placebo, both at rest and following the agility test (40 and 60% vs. 80 and 75%). Furthermore, there was no difference in withdrawal symptoms or total withdrawal scores between conditions.

The effects of acute caffeine ingestion to improve “lower” cognitive functions such as simple reaction time are generally well established [47]. Dosing, ranging from 32 to 300 mg (or roughly 0.5–4 mg·kg^−1^ for a 75 kg individual), has been shown to enhance fundamental aspects of cognitive performance, including attention, vigilance, and reaction time [48,49,50,51]. However, there remains no consensus regarding caffeine’s effects on “higher” cognitive functions, such as problem solving and decision making [52,53]. This may be partly because low to moderate doses of caffeine do not appear to significantly alter sensory functions such as vision or hearing [54]. In the present study, conducted in a population of recreationally active males classified as naïve-mild habitual caffeine users (100.8 ± 47.6 mg·day^−1^; Table 1), acute caffeine supplementation with 300 mg of caffeine ingested ~1 h before testing had no effect on the cognitive test battery employed. We administered a fixed dose, reflecting a practice employed by coaches, that was approximately three times participants’ habitual caffeine intake. Given the wide range in body mass among participants (55–126 kg), we employed ANCOVAs for all outcomes except reactive agility to investigate the potential influence of body mass. When body mass, and therefore relative caffeine dose, was considered, caffeine still had no significant effects on cognitive performance.

The trail-making test assesses a combination of processing speed, sequencing, and visuo-motor skills, with Part B additionally capturing set-shifting [55]. Currently, there is a paucity of research investigating effects of acute caffeine ingestion on performance in the trail-making test A or B, with even fewer studies conducted in the morning using methodologies and sample sizes comparable to those employed in the current study [3,11]. In the current investigation, ingestion of 300 mg of caffeine by individuals with a naïve-mild habitual caffeine consumption (>150 mg·day^−1^) had no effect on morning performance in either the trail-making test Part A or Part B. In contrast, Kim et al. [56] reported that performance in Digit Span tests and trail-making test Part B improved after coffee consumption administered 30 min before testing (time of day was not reported). The authors attributed these cognitive benefits to a reorganization of functional connectivity towards more efficient network properties [56]. Specifically, improvements in executive function were correlated with changes in graph-theoretic measures derived from electroencephalography (EEG), suggesting a shift toward more efficient network organization. Furthermore, executive control is thought to require activation of widespread prefrontal regions in the brain in conjunction with the anterior cingulate cortex [57,58,59]. Supporting the stimulatory effects of caffeine on executive function, caffeine has been shown to increase activity within these brain areas [60,61]. Dopamine is considered a critical neurotransmitter for supporting executive function within these neural networks [62], and caffeine may increase dopamine concentrations through antagonism of adenosine receptors. Consequently, a proposed mechanism by which caffeine enhances executive function involves modulation of dopaminergic pathways connecting the anterior cingulate and prefrontal cortical regions [62]. However, our findings do not support previous studies, which did not account for chronobiological aspects or participants’ body mass and reported beneficial effects of caffeine on executive function and psychomotor speed [57,63,64].

The Stroop test literature, whether using the traditional paper-based method [32,33,34] or computer-delivered methods, suggests caffeine shows little consistent effect on inhibitory control. Although benefits have been observed for attention, vigilance, and certain broader measures of executive function, these improvements do not consistently translate to enhanced Stroop performance [58]. Consistent with our findings, Edwards et al. [65] reported that acute ingestion of 125 and 250 mg of caffeine, assessed approximately 60 min post-ingestion, did not improve performance on the classic Stroop task. However, Hasenfratz et al. [66] demonstrated that a 250 mg dose of caffeine impaired performance on a numerical Stroop task by producing slower responses.

The Auditory-Verbal Learning Test (AVLT) is a memory assessment that evaluates the recall of word lists across both single and multiple trials. We found no differences between the CAFF, PLAC, and NoPill conditions for any AVLT measures. This contrasts with previous findings from our research group, which used the same methodology and morning testing schedule but involved a smaller sample of strength-trained males [11]. Terry and Phifer [67], in a double-blinded, placebo-controlled, independent-groups study involving 19 female and 13 male college students, examined the effects of 100 mg of caffeine. Participants consumed either a caffeine-containing drink or a placebo at 12:30 or 14:00 h and were tested 40 min later. Under the caffeine condition, participants recalled fewer words from the AVLT compared with the control condition, both following a single presentation of the word list and across repeated trials. Furthermore, caffeine consumption was associated with a greater deficit in recalling the middle and end portions of the lists.

The use of agility tests that have a cognitive component, with a sporting context to reflect sport situations, has gained interest over the last decade. Such tests include the reactive Y-test, FitLight/light-based test, reactive t-test, mirror drill, reactive shuttle run, small-sided game test, video/projection test, defensive shuffle reaction test, agility decision course, and dual-task agility test. These assessments require participants to perform tasks of varying complexity, such as sprinting and responding to directional cues, moving towards randomly illuminated targets, changing direction in response to signals, mirroring an opponent’s movement, or making decisions during modified game scenarios. Consequently, they engage in a range of cognitive processes, from lower-order functions such as reaction time to higher-order functions, such as decision-making and executive control. Some agility tests may offer greater ecological validity by more closely replicating sporting situations, such as small-sided games and mirror drills, whereas others provide superior measurement precision and experimental control, including the reactive Y-test and FitLight-based protocols. In contrast, dual-task agility tests and reactive light systems may impose the greatest cognitive demands on participants. Although caffeine appears to enhance lower-order cognitive processes, such as reaction time and attention, evidence for improvements in higher-order decision-making during sport-specific agility tasks remains inconsistent [67,68,69,70,71]. In the current study, caffeine ingestion showed no significant effect on any reactive agility outcome, including the total number of contacts, mean movement time (s), cognitive-motor efficiency (contacts·s^−1^), and variability of movement time (% CV)]. These findings are consistent with the previous research [71]. In contrast, studies conducted at different times of day and employing higher caffeine doses (3–6 mg·kg^−1^) have reported improvements in reaction time and agility performance [68,69]. A limitation of the present study was that we were unable to distinguish reaction time (the interval between stimulus presentation and movement initiation) from movement execution time (the interval between movement initiation and target contact). Consequently, these components were analyzed collectively as movement time.

A significant main effect for light position was observed for both mean movement time and cognitive-motor efficiency. Specifically, lights 1 and 5, which were located furthest from the central reset point (light 3), required longer movement times to react and reach than lights 2 to 4, as would be expected from a mechanical perspective. Consequently, response rates were lower for these distant targets. However, interpretation of the reactive agility findings must be undertaken with caution because of the limited validation and external validity of this assessment. Furthermore, only a subset of the overall sample completed the reactive agility test (27/51 participants), which lowers the statistical power level of the test. Finally, ANCOVA was not performed for the reactive agility outcomes because body mass could potentially influence performance through two separate mechanisms. First, a fixed caffeine dose would result in a lower relative dose (mg·kg^−1^) in heavier participants. Second, greater body mass may independently affect movement speed and agility performance. Including body mass as a covariate could therefore have introduced interpretive complications when assessing caffeine’s effect on reactive agility.

We hypothesized that caffeine would improve morning agility and cognitive performance compared with both the NoPill and PLAC conditions, while also reducing negative mood states and feelings of tiredness. However, we observed no changes in negative mood states or tiredness between conditions. These findings are consistent with previous research suggesting that, under appropriately controlled conditions, the perceived beneficial effects of caffeine on mood and performance are largely attributable to the reversal of withdrawal symptoms following short periods of caffeine abstinence, rather than to a direct psychostimulant effect [72]. In the present study, participants were naïve-mild habitual caffeine consumers and, therefore, were unlikely to experience substantial withdrawal effects following the 24 h abstinence period. Supporting this interpretation, neither the total caffeine withdrawal symptom score nor any withdrawal symptom subscale differed significantly between the CAFF vs. NoPill vs. PLAC conditions (*p* > 0.05, Table 3).

A secondary aim was to measure the rectal core body and mean skin temperatures (T_r_ and T_sk_), as well as mood, caffeine withdrawal symptoms, appetite, and satiety. Mechanistically, caffeine is known to increase sympathetic nervous system activity, circulating catecholamine concentrations, metabolic heat production, and potentially the activation of brown adipose tissue. In the current study, environmental conditions were characterized by a wet-bulb globe temperature of 13–17 °C, dry temperatures of 19–21 °C, relative humidity of 26–44%, barometric pressure of 990–1022 mmHg, and an ambient light intensity of approximately 750 lux. Under these conditions, we observed no differences in T_r_ 60 min after ingestion of 300 mg of caffeine compared with either the no-pill control or placebo conditions (CAFF vs. NoPill: +0.02 °C; CAFF vs. PLAC: +0.04 °C). Similarly, no differences were observed in T_sk_ (CAFF vs. NoPill: −0.38 °C; CAFF vs. PLAC: +0.13 °C). Furthermore, T_r_ remained largely unchanged following the warm-up (Δ = +0.08 °C), whereas T_sk_ decreased (−0.88 °C), reflecting peripheral heat dissipation in response to exercise. However, this effect on T_sk_ was no longer significant when participant body mass was included in the analysis.

Our findings are consistent with previous research demonstrating that acute caffeine doses of 3–6 mg·kg^−1^ administered under thermoneutral conditions produce only small, non-significant increases in core temperature [73,74]. The human body appears capable of dissipating this additional heat through normal thermoeffector responses, thereby preventing substantial elevations in core temperature [74]. Consequently, any changes in cognitive, mood, or agility outcomes observed following caffeine ingestion are unlikely to be mediated by alterations in thermoregulation.

### Limitations

Only young male participants volunteered for the current study, despite opening recruitment to both sexes. We acknowledge that this limits the external validity of our findings, and further research involving female participants and between-sex comparisons is warranted, as biological sex may be a potential factor modulating the effect of caffeine. Participants were recruited from undergraduate and postgraduate student populations; consequently, the age range was relatively narrow (21.4 ± 2.3 years old, Table 1). In addition, we did not measure circulating caffeine concentrations throughout the protocol or genetic polymorphisms associated with caffeine metabolism and sensitivity (e.g., *CYP1A2*, *ADORA2A*). Such measurements would have enabled a more detailed evaluation of inter-individual pharmacokinetic variability, participants’ adherence to the protocol, and the relationship between caffeine and performance. A lack of equipment availability reduced the sample size for the cognitive agility test to 27/51 participants, limiting the power of the analysis. However, there was no evidence of sample bias, as the included and excluded populations had similar characteristics, and there was no order effect; hence, potential residual learning effects were low. We recruited intermediate types and naïve-mild habitual caffeine participants, meaning our findings may not be transferable to extreme chronotypes, females, elite populations, or moderate or high habitual caffeine daily users [75,76]. We registered our study retrospectively on a clinical trial registration; however, we did not change any outcome variables, and a statistical analysis plan was included in the original ethics submission. Further, because we measured a battery of cognitive tests, we acknowledge the possibility of multiplicity bias or Type I error inflation. We also acknowledge that including a group with a high daily caffeine consumption would have added another dimension to the work. Further, there remains a lack of research on higher-order vs. lower-order cognitive function [77]. Finally, our protocol involved participants attending the laboratory in a fasted state. This may limit the generalizability of our findings to athletes who consume breakfast before morning competition due to potential modifications in caffeine pharmacokinetics and resultant effects on cognitive performance.

## 5. Conclusions

Our most important outcome was that ingestion of 300 mg of caffeine 60 min before testing in naïve-mild caffeine consumers had no effect on the battery of cognitive or agility tests spanning a variety of cognitive domains, irrespective of participants’ body mass. Caffeine ingestion did not reduce negative mood states or improve subjective tiredness values, and potential changes in cognitive and agility outcomes are therefore unlikely to be mediated by alterations in thermoregulation. These null findings are consistent with previous literature.

## Figures and Tables

**Table 1:** Physical characteristics (n = 51) and baseline caffeine and daily food intake of the participants (mean ± SD values).

Physical Characteristics and Baseline Caffeine Daily Intake	Mean ± SD Values	
Age (yr)	21.4 ± 2.3	
Height (cm)	179.0 ± 7.8	
Mass (kg)	83.5 ± 16.1	
Chronotype	32.5 ± 5.0	
Languid/Vigorous	43.9 ± 6.2	
Flex/Rigidity	44.1 ± 6.1	
Athletic level (1–5)	2.1 ± 0.8	
Habitual sleep (decimal h)	8.4 ± 0.6 h	
Pittsburgh sleep questionnaire	3.2 ± 1.0	
Caffeine intake from questionnaire (mg)	100.8 ± 47.6	
**Baseline Daily Food Intake**	**Participants Intake**	RDA *
Daily calories (kcal)	**2222 ± 474**	**2500 (*p* < 0.0001)**
Fats (g)	88 ± 36	97 (*p* = 0.564)
Protein (g)	**154 ± 44**	**55.5 (*p* < 0.001)**
Carbohydrates (g)	**179 ± 55**	**330 *(p* < 0.001)**
Zinc (mg)	9.9 ± 4.6	11.0 (*p* = 0.093)
Magnesium (mg)	**247 ± 98**	**400 (*p* < 0.0001)**
Vitamin B6 (mg)	**2.7 ± 1.7**	**1.4 (*p* < 0.0001)**
Water intake (mL)	**3459 ± 1220**	**2000 (*p* < 0.0001)**

* Denotes recommended daily values (RDA) from Nutrition Science Team [23]. Bold values denote significance of participants’ *p*-values compared to RDA from a paired *t*-test.

**Table 2:** Mean ± SD values for main effect for condition (NoPill, CAFF, or PLAC) for all measures. Main effect for measure and interactions (condition by measure), as well as covariates for mass, to investigate if main effects differ according to body mass. *p*-values and partial eta squared values (η^2^_p_) are given for rectal and mean skin temperature, all variables associated with the reactive agility test [total number of contacts, mean movement time (s), cognitive-motor efficiency (contacts·s^−1^), variability of movement time (% CV; coefficient of variation)] and hunger and satiety values. The reactive agility test was not analyzed with consideration for mass.

Variable	NoPill	CAFF	PLAC	Significance Condition/Mass Interaction (*p*, η^2^_p_)	Significance of Measure (Pre–Post; Lights)/Mass Interaction (*p*, η^2^_p_)	Condition by Measure Interactions/Mass Interaction (*p*, η^2^_p_)
*Temperature* (n = 51)						
Rectal temperature (°C)	36.83 ± 0.50	36.89 ± 0.45	36.82 ± 0.42	*p* = 0.219, 0.011; *p* = 0.215, 0.015	*p* = 0.087, 0.133; *p* = 0.503, 0.005	*p* = 0.338, 0.023; *p* = 0.544, 0.031
Mean skin temperature (°C)	30.54 ± 1.05 *	29.73 ± 1.50	29.64 ± 0.96	*p* = 0.107, 0.078; *p* = 0.356, 0.098	***p*** = **0.007**, **0.982**; *p* = 0.836, 0.097	*p* = 0.331, 0.034; *p* = 0.716, 0.093
*Reactive Agility Test* (n = 27)						
Total number of contacts	23.80 ± 7.98	23.20 ± 6.84	23.73 ± 6.78	*p* = 0.149, 0.072	*p* = 0.146, 0.063	*p* = 0.095, 0.062
Mean movement time (s)	0.81 ± 0.18	0.78 ± 0.16	0.77 ± 0.15	*p* = 0.113, 0.081	***p*** < **0.001**, **0.508**	*p* = 0.188, 0.053
Cognitive-motor efficiency (contacts·s^−1^)	23.80 ± 7.98	23.20 ± 6.84	23.73 ± 6.78	*p* = 0.096, 0.086	***p*** < **0.001**, **0.582**	*p* = 0.535, 0.026
Variability of Movement Time (% CV)	0.81 ± 0.18	0.78 ± 0.16	0.77 ± 0.15	*p* = 0.075, 0.095	*p* = 0.522, 0.030	*p* = 0.219, 0.051
*Hunger and Satiety Scales* (n = 51)						
I feel hungry	5.0 ± 2.2	5.0 ± 2.6	4.7 ± 2.6	*p* = 0.580, 0.011; *p* = 0.408, 0.008		
My stomach feels full	2.9 ± 1.8	3.2 ± 2.1	2.7 ± 1.7	*p* = 0.920, 0.002; *p* = 0.802, 0.004		
I have a desire to eat something savoury	4.4 ± 2.7	4.7 ± 2.7	4.1 ± 2.7	*p* = 0.743, 0.006; *p* = 0.549, 0.012		
I have a desire to eat something sweet	4.1 ± 2.7	4.5 ± 2.8	4.0 ± 2.2	*p* = 0.086, 0.049; *p* = 0.051, 0.059		
I feel physically tired	4.1 ± 2.6	4.4 ± 2.6	4.5 ± 2.2	*p* = 0.831, 0.004; *p* = 0.688, 0.008		
I feel sleepy/drowsy/half awake	3.9 ± 2.4	4.0 ± 2.5	4.2 ± 2.6	*p* = 0.434, 0.017; *p* = 0.311, 0.024		
I feel energetic/active/lively	4.6 ± 1.9	4.7 ± 2.3	4.4 ± 2.0	*p* = 0.937, 0.001; *p* = 0.953, 0.011		
I feel lethargic/sluggish	3.8 ± 2.6	3.7 ± 2.8	4.0 ± 2.7	*p* = 0.977, 0.000; *p* = 0.949, 0.001		

Bold *p*-values indicate significance (*p* < 0.05).

**Table 3:** Mean ± SD values for main effect for cognitive performance tests (‘trail-making’ [A, B], ‘Rey’s auditory verbal learning test (RAVLT)’ and ‘Stroop’) with interaction of mass, in the three experimental conditions (no-pill control [NoPill], caffeine [CAFF], and placebo ingestion [PLAC]); *p*-values and partial eta squared values (η^2^_p_) are given.

Variables (n = 51)	NoPill	CAFF	PLAC	Main Effect for Condition/Interaction Mass
*Cognitive tests*				
Trail-making test A (s)	14.2 ± 3.9	14.2 ± 3.8	14.1 ± 3.7	*p* = 0.832, 0.004; *p* = 0.842, 0.004
Trail-making test B (s)	33.7 ± 14.8	30.1 ± 11.1	35.4 ± 12.8	*p* = 0.095, 0.049; *p* = 0.117, 0.044
Trail-making test B-A (s)	19.5 ± 14.4	15.9 ± 9.2	21.3 ± 11.6	*p* = 0.078, 0.052; *p* = 0.086, 0.050
RAVLT—total number	53.8 ± 12.1	54.3 ± 11.7	54.1 ± 11.7	*p* = 0.877, 0.003; *p* = 0.844, 0.003
RAVLT—distractions	6.7 ± 2.4	6.8 ± 2.8	6.8 ± 2.8	*p* = 0.678, 0.004; *p* = 0.709, 0.003
RAVLT—retention	10.9 ± 3.5	10.9 ± 3.2	11.1 ± 3.2	*p* = 0.308, 0.024; *p* = 0.314, 0.023
*Stroop*				
Colours, not words—number	64.1 ± 12.5	65.4 ± 12.4	65.4 ± 11.7	*p* = 0.338, 0.021; *p* = 0.349, 0.020
Colours, not words—error	0.7 ± 1.3	0.6 ± 1.0	0.6 ± 1.0	*p* = 0.932, 0.001; *p* = 0.977, 0.000
Colours interference—number	14.7 ± 7.0	15.1 ± 9.9	14.8 ± 6.3	*p* = 0.892, 0.002; *p* = 0.869, 0.002
Words, not colours—number	108.4 ± 26.5	105.7 ± 29.7	112.0 ± 25.6	*p* = 0.929, 0.001; *p* = 0.850, 0.003
Words, not colours—error	0.6 ± 1.0	0.6 ± 0.9	0.6 ± 1.0	*p* = 0.567, 0.012; *p* = 0.536, 0.013
Word interference—number	6.3 ± 5.9	5.8 ± 8.7	4.6 ± 5.0	*p* = 0.280, 0.025; *p* = 0.366, 0.020

**Table 4:** Mean (±SD) values for sleep questions, tiredness, and alertness, perceived onset of mood scores (POMS), and caffeine withdrawal symptom scores for three conditions (NoPill, CAFF, or PLAC), with interaction for mass. Statistics are given [*p*-values and partial eta squared values (η^2^_p_)].

Variables	NoPill	CAFF	PLAC	Main Effect for Condition/Interaction Mass
*Sleep questions*				
Ease to sleep	0.2 ± 2.6	0.7 ± 2.5	0.1 ± 2.4	*p* = 0.587, 0.011; *p* = 0.528, 0.013
Get to sleep	0.1 ± 2.0	−0.3 ± 2.2	0.5 ± 2.6	*p* = 0.413, 0.018; *p* = 0.444, 0.016
Well Slept	−0.1 ± 2.4	0.3 ± 2.2	0.7 ± 2.4	*p* = 0.303, 0.024; *p* = 0.315, 0.023
Waking time	−2.1 ± 1.8	−2.2 ± 1.9	−2.1 ± 1.6	*p* = 0.670, 0.008; *p* = 0.701, 0.007
Alertness 15 min after waking	−0.1 ± 1.9	0.0 ± 2.3	−0.2 ± 1.9	*p* = 0.681, 0.008; *p* = 0.625, 0.010
Tiredness (0–10 VAS)	4.5 ± 2.2	4.6 ± 2.6	4.9 ± 2.5	*p* = 0.795, 0.005; *p* = 0.720, 0.007
Alertness (0–10 VAS)	5.5 ± 1.7	5.5 ± 1.9	5.2 ± 2.0	*p* = 0.844, 0.003; *p* = 0.940, 0.001
Stanford Sleep Questionnaire	2.8 ± 1.1	2.5 ± 1.0	2.9 ± 0.9	*p* = 0.779, 0.005; *p* = 0.624, 0.009
*POMS*				
Mood State—Vigour	6.4 ± 3.0	6.4 ± 3.7	6.0 ± 3.4	*p* = 0.230, 0.030; *p* = 0.275, 0.026
Mood State—Anger	0.7 ± 1.5	0.8 ± 1.9	1.0 ± 2.3	*p* = 0.557, 0.011; *p* = 0.498, 0.013
Mood State—Tension	0.7 ± 1.4	0.9 ± 1.5	1.0 ± 1.7	*p* = 0.694, 0.006; *p* = 0.786, 0.004
Mood State—Calm	7.9 ± 2.8	7.6 ± 3.0	8.0 ± 2.4	*p* = 0.865, 0.003; *p* = 0.927, 0.002
Mood State—Happiness	6.9 ± 2.7	7.0 ± 3.1	7.2 ± 2.8	*p* = 0.590, 0.011; *p* = 0.581, 0.011
Mood State—Confusion	0.9 ± 1.8	1.3 ± 2.0	2.0 ± 3.2	*p* = 0.592, 0.010; *p* = 0.861, 0.002
Mood State—Depression	0.9 ± 2.2	0.8 ± 1.8	1.0 ± 1.9	*p* = 0.668, 0.008; *p* = 0.584, 0.011
Mood State—Fatigue	4.6 ± 3.6	4.7 ± 3.9	5.3 ± 4.0	*p* = 0.949, 0.001; *p* = 0.959, 0.001
*Caffeine Withdrawal symptoms*				
Total scores	26.7 ± 9.4	26.8 ± 10.2	27.5 ± 9.6	*p* = 0.058, 0.056; *p* = 0.062, 0.063
Fatigue/drowsiness	4.6 ± 3.1	4.4 ± 3.0	4.6 ± 3.1	*p* = 0.119, 0.043; *p* = 0.099, 0.047
Low alertness/difficulty concentrating	8.6 ± 2.6	8.9 ± 3.1	9.0 ± 2.5	*p* = 0.385, 0.019; *p* = 0.329, 0.022
Mood disturbances	2.0 ± 2.4	2.2 ± 2.3	1.8 ± 1.9	*p* = 0.196, 0.033; *p* = 0.256, 0.027
Low sociability/motivation to work	8.9 ± 3.8	8.8 ± 4.3	8.8 ± 4.1	*p* = 0.438, 0.017; *p* = 0.413, 0.018
Nausea/upset stomach	0.4 ± 0.7	0.5 ± 0.9	0.5 ± 1.0	*p* = 0.592, 0.011; *p* = 0.451, 0.016
Flu-like feelings	1.3 ± 1.6	1.2 ± 1.3	1.4 ± 1.7	*p* = 0.576, 0.011; *p* = 0.476, 0.015
Headache	0.4 ± 0.7	0.3 ± 0.6	0.4 ± 0.7	*p* = 0.833, 0.003; *p* = 0.787, 0.004
Questions 21–32	2.2 ± 2.7	2.3 ± 3.1	1.9 ± 2.7	*p* = 0.204, 0.032; *p* = 0.284, 0.025

## Data Availability

The data presented in this study are available on request from the corresponding author due to privacy or ethical restrictions.

## Supplementary Materials

The following supporting information can be downloaded at: https://www.mdpi.com/article/10.3390/nu18172771/s1, Figure S1: CONSORT 2025 checklist of information to include when reporting a randomised trial [21]; Figure S2: CONSORT 2025 Flow Diagram of the progress through the phases of a randomised trial of two groups (that is, enrolment, intervention allocation, follow-up, and data analysis) [21].
